# Single-dose HPV vaccination efficacy among adolescent girls and young women in Kenya (the KEN SHE Study): study protocol for a randomized controlled trial

**DOI:** 10.1186/s13063-021-05608-8

**Published:** 2021-09-27

**Authors:** Ruanne V. Barnabas, Elizabeth R. Brown, Maricianah Onono, Elizabeth A. Bukusi, Betty Njoroge, Rachel L. Winer, Deborah Donnell, Denise Galloway, Stephen Cherne, Kate Heller, Hannah Leingang, Susan Morrison, Elena Rechkina, R. Scott McClelland, Jared M. Baeten, Connie Celum, Nelly Mugo, for the KEN SHE Study Team

**Affiliations:** 1 Department of Global Health, University of Washington, Seattle, USA; 2 Division of Allergy and Infectious Diseases, University of Washington, Seattle, USA; 3 Department of Epidemiology, University of Washington, Seattle, USA; 4 Vaccine and Infectious Disease Division, Fred Hutchinson Cancer Research Center, Seattle, USA; 5 Department of Biostatistics, University of Washington, Seattle, USA; 6 Human Biology Division, Fred Hutchinson Cancer Research Center, Seattle, USA; 7 Kenya Medical Research Institute, Nairobi, Kenya; 8 Department of Laboratory Medicine and Pathology, University of Washington, Seattle, USA

**Keywords:** Human papillomavirus, Randomized controlled trial, Single-dose vaccination, Reduced dose schedule, Multi-age cohort

## Abstract

**Background:**

HPV infection is the primary cause of cervical cancer, a leading cause of cancer among women in Kenya and many sub-Saharan African countries. High coverage of HPV vaccination is a World Health Organization priority to eliminate cervical cancer globally, but vaccine supply and logistics limit widespread implementation of the current two or three dose HPV vaccine schedule.

**Methods:**

We are conducting an individual randomized controlled trial to evaluate whether a single dose of the bivalent (HPV 16/18) or nonavalent (HPV 16/18/31/33/45/52/58/6/11) HPV vaccine prevents persistent HPV infection, a surrogate marker for precancerous lesions and cervical cancer. The primary objective is to compare the efficacy of immediate, single-dose bivalent or nonavalent vaccination with delayed HPV vaccination. Kenyan women age 15–20 years old are randomized to immediate bivalent HPV and delayed meningococcal vaccine (group 1), immediate nonavalent HPV vaccine and delayed meningococcal vaccine (group 2), or immediate meningococcal vaccine and delayed HPV vaccine (group 3) with 36 months of follow-up. The primary outcome is persistent vaccine-type HPV infection by month 18 and by month 36 for the final durability outcome. The secondary objectives include to (1) evaluate non-inferiority of antibody titers among girls and adolescents (age 9 to 14 years) from another Tanzanian study, the DoRIS Study (NCT02834637), compared to KEN SHE Study participants; (2) assess the memory B cell immune response at months 36 and 37; and (3) estimate cost-effectiveness using the trial results and health economic models.

**Discussion:**

This study will evaluate single-dose HPV vaccine efficacy in Africa and has the potential to guide public health policy and increase HPV vaccine coverage. The secondary aims will assess generalizability of the trial results by evaluating immunobridging from younger ages, durability of the immune response, and the long-term health benefits and cost of single-dose HPV vaccine delivery.

**Trial registration:**

ClinicalTrials.gov NCT03675256. Registered on September 18, 2018

**Supplementary Information:**

The online version contains supplementary material available at 10.1186/s13063-021-05608-8.

## Administrative information

Note: the numbers in curly brackets in this protocol refer to the SPIRIT checklist item numbers. The order of the items has been modified to group similar items (see http://www.equator-network.org/reporting-guidelines/spirit-2013-statement-defining-standard-protocol-items-for-clinical-trials/).

**Table:** 

Title {1}	Single-dose HPV vaccination efficacy among adolescent girls and young women in Kenya (the KEN SHE Study): Study protocol for a randomized controlled trial
Trial registration {2a and 2b}.	NCT03675256 https://clinicaltrials.gov/ct2/show/NCT03675256 Date registered: First Posted: September 18, 2018
Protocol version {3}	2.0, January 7th, 2021
Funding {2}	Bill & Melinda Gates Foundation (OPP1188693) and the University of Washington King K. Holmes Endowed Professorship in STDs and AIDS
Author details {5a}	^1^Department of Global Health, ^2^Division of Allergy and Infectious Diseases, ^3^Department of Epidemiology, ^4^Department of Biostatistics, ^5^Department of Laboratory Medicine and Pathology, University of Washington, Seattle, USA; ^6^Vaccine and Infectious Disease Division, Human Biology Division, Fred Hutchinson Cancer Research Center, Seattle, USA; ^8^Kenya Medical Research Institute, Kenya
Name and contact information for the trial sponsor {5b}	Trial Sponsor: University of Washington Contact: Office of Sponsored Programs Email: osp@uw.edu
Role of sponsor {5c}	The content is solely the responsibility of the authors and does not necessarily represent the views, decisions, or policies of the institutions with which they are affiliated or the KEN SHE Study funders. The funders had no role in study design; and have no role in the data collection, analysis, and interpretation; writing of the protocol paper; or in the decision to submit for publication. The corresponding author had final responsibility for the decision to submit the protocol paper for publication.

## Introduction

### Background and rationale {6a}

Cervical cancer, caused primarily by the human papillomavirus (HPV), is a leading cause of incident cancer cases among women in Africa [1]. HPV vaccines have high efficacy and prevent almost 100% of vaccine-type specific HPV infection and disease and dramatically reduce the risk of cervical cancer by > 90% at the individual level [2, 3]. However, in sub-Saharan Africa where more than 80% of cervical cancer cases occur, HPV vaccine coverage remains low and cervical cancer screening and treatment is limited [4]. Currently, three HPV vaccines are licensed, which target high-risk HPV types that cause 70% of cancers (HPV 16/18) and low-risk HPV types that cause genital warts (HPV 6/11); the bivalent vaccine protects against HPV 16/18 (Cervarix®), the quadrivalent vaccine against HPV 16/18/6/11 (Gardasil®), and the nonavalent vaccine against nine HPV genotypes including seven high-risk HPV types (HPV 16/18/31/33/45/52/58/6/11 - Gardasil-9®) [5]. All three vaccines are licensed for two or three doses dependent on the age of the client at vaccination. In countries that have achieved high HPV vaccine coverage at population level and that use the multi-age cohort vaccination approach of immunizing 9–26 year olds, HPV-associated moderate or severe precancerous lesions have decreased by almost 100% compared to countries with single-cohort vaccination (e.g., age 9–10 years) or low routine vaccination demonstrating the substantial impact of widespread HPV vaccination on precancerous lesions [6].

Kenya’s national immunization program, launched in October 2019, offers two doses of the HPV vaccine to 9–10-year-old girls. Further, the Global Alliance for Vaccines and Immunization (GAVI) endorses multi-age cohort vaccination to age 14 years [7]. A gap exists in prevention strategies for young women age 15–20 years, for whom three doses of the HPV vaccine is currently recommended, and the age at which HPV acquisition is highest. Preliminary observation evidence suggests that efficacy of single-dose of HPV vaccination is equivalent to two or three doses [8–12]. Addressing this evidence gap on the efficacy and durability of single-dose HPV vaccine among young women in Africa, particularly as a multi-age cohort vaccination strategy for women age 15–20 years, would simplify the costs of logistics of effective prevention for this priority population. Importantly, the multi-age cohort approach would in the short term provide vaccine protection to adolescents and girls who are not eligible for routine vaccination.

Demonstration projects support the feasibility of high coverage of single-dose HPV vaccination in Kenya. Between 2013 and 2015 and 2016–2018, demonstration projects of the quadrivalent HPV 16/18/6/11 vaccine offered to girls age 9–10 years in a school-based setting achieved > 85% coverage of the first dose with lower coverage of subsequent doses; 64% uptake of the second dose and 39% of the third doses [13, 14]. Furthermore, the current national single-cohort (9- and 10-year-old girls) HPV vaccination program is clinic based and coverage has been lower and affected by the COVID-19 pandemic. The key barriers for transitioning from a single-cohort immunization program to a multi-cohort program are the total costs and logistics associated with the two or three dose vaccine schedules.

The rationale for a single-dose HPV vaccine study among young Kenyan women age 15–20 years is threefold. First, cervical cancer is the leading cause of new cancer cases among women in Africa and HPV vaccine scale-up has the potential to dramatically decrease cervical cancer cases. Second, replacing the two or three dose HPV vaccine schedule with the single-dose strategy, efficacy of which is supported by observational data [8–11], would be transformative from a logistics and cost perspective. Lastly, a gap exists within the current HPV vaccination landscape to warrant a focus on reaching young women who are older than the current recommended age for vaccination (9–14 year olds) in national programs for catch-up vaccination and facilitate a rapid decrease in precancerous lesions. Kenya has the scientific background, experience, and expertise to conduct the study and the political will to capitalize on the results, if positive, within their national immunization program. Thus, we are conducting a randomized clinical trial to test the efficacy of single-dose HPV vaccination among girls and adolescents in Kenya to determine the optimal strategy for HPV vaccination.

## Objectives {17}

SPIRIT guidance: specific objectives or hypotheses.

### Primary objectives

1. Test the efficacy of immediate single-dose bivalent or nonavalent HPV vaccination to prevent incident persistent HPV 16/18 infection compared to delayed HPV vaccination for young women age 15–20 years
2. Test the efficacy of immediate single-dose nonavalent HPV vaccination to prevent incident persistent HPV 16/18/31/33/45/52/58 infection compared to delayed HPV vaccination for young women age 15–20 years

#### Hypotheses

1. Single-dose bivalent or nonavalent HPV vaccination will prevent 75% of combined incident persistent HPV-16/18 infections among young women who are HPV 16/18 naïve at enrollment*.*
2. Single-dose nonavalent HPV vaccination will prevent 75% of combined incident persistent HPV 16/18/31/33/45/52/58 among young women who are 16/18/31/33/45/52/58 naïve at enrollment.

### Secondary objectives

1. Determine whether vaccine-type HPV antibody responses after single-dose bivalent or nonavalent vaccination are noninferior in 9–14-year-old girls versus 15–20-year-old young women
2. Assess cost, cost-effectiveness, and budget impact of single-dose HPV vaccination to support implementation strategies for single-dose HPV vaccination following WHO recommendation in high cervical cancer burden settings
3. Evaluate B cell markers as a proxy for central immune memory following single-dose bivalent and nonavalent vaccination
4. Assess vaccine efficacy with the exclusion of infections first detected at month 6, as these may represent prevalent infections not detected at baseline rather than incident infections

#### Hypotheses

1. Antibody responses to vaccine-type HPV after receiving a single-dose of bivalent or nonavalent HPV vaccine will be noninferior in 9–14-year-old girls and adolescents compared to 15-20-year-old adolescents and young women.
2. Single-dose bivalent and nonavalent HPV vaccination will be cost-effective per cervical cancer case averted*.*
3. Single-dose HPV vaccination will induce potent memory with B cells producing neutralizing antibodies.
4. Single-dose bivalent or nonavalent HPV vaccination will prevent 75% of combined incident persistent vaccine-type HPV infections among young women who are vaccine-type HPV naïve at enrollment, month 3, and month 6*.*

## Trial design {18}

The KEN SHE Study is an individual, blinded, prospective randomized trial among 2250 adolescent girls and young women with potential exposure to high-risk HPV in Kenya to test the efficacy of single-dose HPV vaccination to prevent incident persistent HPV infection. This is a superiority trial. Participants are randomized 1:1:1 to either (1) immediate nonavalent HPV vaccination and delayed meningococcal vaccination, (2) immediate bivalent HPV vaccination and delayed meningococcal vaccination, or (3) immediate meningococcal HPV vaccination and delayed HPV vaccination.

## Methods: participants, interventions, and outcomes

### Study setting {19}

The study is conducted at three locations in Kenya: (1) Thika, a town in Kiambu County, northeast of Nairobi (Kenya Medical Research Institute (KEMRI) Thika), (2) KEMRI clinic at the Center of Clinical Research (CCR) in Nairobi (KEMRI Nairobi), and (3) Kisumu, in western Kenya (KEMRI Kisumu). The Nairobi site is urban, while the Thika and Kisumu sites are peri-urban. Participants are young, HIV-negative women, age 15–20 years, who report 1–5 sex partners in their lifetime. We estimated that approximately 9000 women would be screened to enroll 2250 eligible participants for the study.

### Eligibility criteria {10}

The study clinical personnel reviewed the screening questionnaire, clinical history, and the HIV testing results to determine study eligibility.

The study eligibility criteria are:

- Female sex assigned at birth
- Age 15 to 20 years
- Sexually active: defined as history of 1-5 lifetime sex partners
- Resident within study area without plans to move away in the next 37 months

Ineligibility criteria for the study are:

- Positive HIV rapid serologic test result
- History of HPV vaccination
- Allergies to vaccine components or latex
- Current pregnancy
- Hysterectomy
- Autoimmune, degenerative, or genetic diseases
- Investigator discretion

Participants receive their screening results and counseling regarding clinical management as indicated. Participants who are eligible receive an appointment for follow-up at the study clinic to conduct the enrollment procedures.

### Who will take informed consent? {26a}

The study is implemented according to the principles stated in the Declaration of Helsinki and the principles of the International Conference on Harmonization (ICH) Guideline for Good Clinical Practice (GCP). Study staff from all collaborating institutions are trained in GCP prior to study start. The laboratory staff are trained in Good Clinical Laboratory Practice (GCLP). The study staff conduct the informed consent or assent procedures.

All participants, and their parents/guardians in the case of minors, go through an informed assent/consent process. Information is provided on sexual and reproductive health services including cervical cancer prevention services available outside the study. All potential clients read or have read to them the consent form by the study team. The consent form is available in both the local language and English, and participants select which language they prefer to be consented in. Each participant, and their parent/guardian, has an opportunity to ask questions about study participation. Once a participant agrees to participate in the study, the study staff obtains written consent. After consenting to participate, a participant can voluntarily withdraw from the study at any time and can choose not to have their responses submitted to the study team.

### Additional consent provisions for collection and use of participant data and biological specimens {26b}

Participants separately provide consent for storage of blood and genitourinary swabs at the Thika, Nairobi, and Kisumu KEMRI sites and at the University of Washington and the Fred Hutchinson Cancer Research Center for future research. Specifically, participants consent to samples being shipped to a central laboratory at the National Cancer Institute for immunobridging analyses and comparison of other trial results to the KEN SHE Study findings. Further, samples and data could be used for research related to HPV vaccines, HPV, bacterial vaginosis (BV), HIV, HIV-related diseases, and other sexually transmitted infections (STIs).

## Interventions

### Explanation for the choice of comparators {6b}

Kenya is part of the meningitis belt, with meningococcal vaccination recommended for adolescents and young persons entering the first year of university/college. During large scale *Neisseria meningitidis* outbreaks which occur every 5–12 years, meningitis incidence can be as high as 1000 cases per 100,000 population per year [15]. Timing of meningococcal vaccine deployment is based on current outbreaks, but early vaccination would ensure antibody protection during future epidemics. Study participants will have access to an effective vaccine to prevent meningococcal disease. The meningococcal vaccine is a registered and approved vaccine in the national program and is safe and potentially beneficial to participants. The meningococcal vaccine is expected to have no cross-reaction with HPV vaccine, thus serving as akin to a placebo control, but offering protection against meningococcal disease as a clinical benefit.

### Intervention description {11a}

#### Eligibility confirmation

Prior to randomization, eligibility is rechecked and documented on the case report form (CRF). If eligible, the participant is randomized and receives the first dose of the vaccination. Participants who are not eligible are referred to care as needed and the reasons for exclusion are explained to them.

#### History and examination procedures

Study staff take a detailed medical and sexual reproductive health history including symptoms of sexually transmitted infections (STIs). The procedures for the pelvic and speculum exams are explained to the participants/guardians, questions answered, and participants may choose to have a chaperone present for the examination. A pelvic examination is done and genital specimens are collection. Baseline visit procedures and samples are collected as follows: external genitalia are inspected for genital warts and the results recorded on the CRF; an external genitalia swab (labial/vulvar/perineal) is collected for HPV DNA; on speculum examination a lateral vaginal and a cervical swab for HPV DNA, Pap smear, and samples for other STI testing (including chlamydia and gonorrhea, trichomoniasis, and bacterial vaginosis) are collected; and 10 mL of blood is collected for HPV antibodies and syphilis testing.

#### Comprehensive sexual and reproductive health services

Comprehensive sexual and reproductive health services are offered to participants at every visit. These include pregnancy testing, counseling, and provision of contraceptive services; testing, treatment, and counseling for sexually transmitted infections; and HIV testing, counseling, and oral pre-exposure prophylaxis (PrEP) to prevent HIV acquisition. PrEP provision follows the Kenyan national guidelines, with medication available from the Ministry of Health, and referral to other PrEP providers in Thika, Nairobi, and Kisumu for participants who would prefer to access PrEP services outside the study.

Throughout the study, cervical cancer screening, with appropriate counseling, management and referral if indicated, is provided to participants in real time. Management follows local guidelines.

#### Vaccination

At the end of the enrollment visit, participants receive their masked vaccination according to their enrollment arm (bivalent HPV vaccine, nonavalent HPV vaccine, or meningococcal vaccine).

Study vaccines were administered by intramuscular injection into the deltoid region of the upper arm. Participants were monitored for 30 min after vaccine administration in the study clinic and serious adverse events (SAEs) recorded. Participants received counseling on the expected reaction to vaccinations, information on self-care, and were asked to report new local and systemic symptoms that arise after they leave the study clinic. Counseling is provided emphasizing that participants and staff do not know the randomization arm.

### Criteria for discontinuing or modifying allocated interventions {11b}

Since the intervention is a single-dose vaccination, once the immunization is administered it cannot be discontinued or modified. Participants are counseled to report vaccinations outside the study. Participants may choose to receive HPV and meningococcal vaccinations outside the study and those are recorded in the data and accounted for in the analysis.

### Strategies to improve adherence to interventions {11c}

Since the vaccine is administered as a single dose, retention to ensure complete data and support well powered analyses are key to assess the efficacy of single-dose HPV vaccination. The sites’ retention strategies are based on personalized attention, empathy, and study staff support for the adolescents and young women throughout their study participation.

### Relevant concomitant care permitted or prohibited during the trial {11d}

The clinical trial provides scheduled Pap smear screening at enrolment and exit visits. Comprehensive sexual and reproductive health services are provided quarterly, detailed in study procedures below. Participants are encouraged to report HPV vaccination outside the study to the study team.

### Provisions for post-trial care {30}

Participants will receive the recommended WHO HPV vaccination schedule at the end of the study, dependent on the study results. Participants who did not receive the meningococcal vaccination will receive that immunization. Participants with abnormal Pap smears at study exit will be followed until management is complete and no further interventions are required.

## Outcomes {12}

### Primary outcomes

1. Percent reduction in the incidence rate of persistent bivalent vaccine HPV types (i.e., HPV 16 and/or HPV 18) per 100 women-years at risk, at month 18 for the primary analysis and at month 36 for the final analysis.
2. Percent reduction in the incidence rate of persistent nonavalent vaccine HPV types (i.e., HPV 16, 18, 31, 33, 45, 52, and/or 58) per 100 women-years at risk, at month 18 for the primary analysis and at month 36 for the final analysis.

Persistent high-risk HPV infection, defined as HPV infection 6 months apart, is a surrogate marker for dysplastic lesions and progression to cervical cancer.

### Secondary outcomes

1. The proportion of participants with vaccine type specific HPV antibody responses after vaccination. Antibody titers will be measured 1 month after vaccination to reflect the peak response and 24 months after vaccination to reflect the plateau phase antibody titer. Antibody titer will capture the proportion of participants who successfully develop type-specific HPV antibodies post vaccination.
2. B cell markers, as a measure of long-term immunity, will be measured at months 1 and 37 to capture the early response and the plateau phase response. B cell markers are a measure of the long-term memory of the immune response, separate from the antibody titers.
3. The incremental cost of vaccine delivery will be estimated at the month 36 vaccination.
4. The percent reduction in the incidence rate of persistent bivalent HPV vaccine types (i.e., HPV 16 and/or 18) and persistent nonavalent HPV vaccine types (HPV 16, 18, 31, 33, 45, 52, and/or 58) infection per 100 women-years at risk, excluding participants with infections detected at month 6, at month 18 for the primary analysis and at month 36 for the final analysis. Exclusion of infections detected at month 6 will exclude potential infections that were not detected at enrolment.

## Participant timeline {13}

**Fig. 1:**
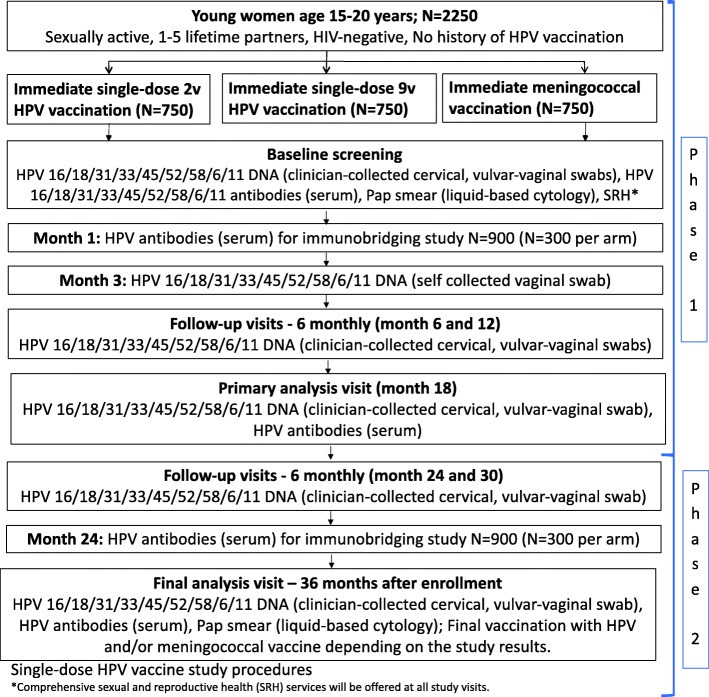
Participant enrollment, intervention, and assessment

The timeline for participant enrolment, intervention, and assessment is shown in Table 1 and Fig. 1.

**Table 1:** Study procedures

				Study period													
	Screening	Enrollment	Allocation	Post-allocation												Close-out	
Timepoint**	***-t***_***2***_	***-t***_***1***_	**0**	***t***_***1***_ ***(m1)***	***t***_***2***_ ***(m3)***	***t***_***3***_ ***(m6)***	***t***_***4***_ ***(m9)***	***t***_***5***_ ***(m12)***	***t***_***6***_ ***(m15)***	***t***_***7***_ ***(m18)***	***t***_***8***_ ***(m21)***	***t***_***9***_ ***(m24)***	***t***_***10***_ ***(m27)***	***t***_***11***_ ***(m30)***	***t***_***12***_ ***(m33)***	***t***_***13***_ ***(m36)***	***t***_***14***_ ***(m37)***
**Screening**																	
**Eligibility screen**	X																
**Informed consent/assent**	X																
**enrolment:**																	
**Eligibility screen**		X															
**Informed consent/assent**		X															
***Demographics & enrollment questionnaire***		X															
***Randomization***			X														
**Masked vaccination**			X														
**Follow-up**																	
**Follow-up questionnaire**				X^a^	X	X	X	X	X	X	X	X	X	X	X		
**Record for post vaccination AEs**				X^a,e^	X^e^												
**Specimen collection**																	
***External genital swab for HPV DNA***		X															
***Self-collected vaginal swab for HPV DNA***					X												
***Cervical swab for HPV DNA***		X				X^f^		X^f^		X^f^		X^f^		X^f^		X^f^	
***Provider-collected vaginal swab for HPV DNA***		X				X^f^		X^f^		X^f^		X^f^		X^f^		X^f^	
***Serum for HPV Antibodies (5 mL whole blood)***		X		X^a,c^						X		X^c^				X	
***Collect PBMC for B cell study (40 mL)***				X^a,d^												X^d^	X^b,d^
***Pap smear***		X														X	
***Rapid HIV test- finger prick or arm (2 mL whole blood)***	X	X			X	X	X	X	X	X	X	X	X	X	X	X	
***Urine for pregnancy test (20 mL)***	X	X			X	X	X	X	X	X	X	X	X	X	X	X	
***STI testing (cervical/vaginal swab) for CT/GC NAATS testing***^***g***^		X				X		X		X		X		X		X	
***STI testing (cervical/vaginal swab) for T. Vaginalis NAATS testing***^***g***^		X														X	
***STI testing (cotton swab) for Bacterial Vaginosis***^***g***^		X				X		X		X		X		X		X	
***STI testing (5 mL whole blood) Serum for syphilis***^***g***^		X				X		X		X		X		X		X	
***STI testing (5 mL whole blood) serum for HSV-2***^***g***^		X														X	
**Assays**																	
***HPV DNA***		X			X	X		X		X		X		X		X	
***HPV Antibody Luminex***		X		X^a,c^						X		X^c^				X	
***Memory B cell***				X^a,d^												X	X^b,d^
***Pap smear***		X														X	
***HIV rapid***	X	X			X	X	X	X	X	X	X	X	X	X	X	X	
***Pregnancy rapid***	X	X			X	X	X	X	X	X	X	X	X	X	X	X	
***CT/GC***		X				X		X		X		X		X		X	
***T . Vaginalis***		X														X	
***Bacterial Vaginosis***		X				X		X		X		X		X		X	
***Syphilis***		X				X		X		X		X		X		X	
***HSV-2***		X														X	
**Standard procedures**																	
***Review medical history***		X		X^a^	X	X	X	X	X	X	X	X	X	X	X	X	
***Offer contraception***	X	X		X^a^	X	X	X	X	X	X	X	X	X	X	X	X	X
**Offer PrEP**	X	X		X^a^	X	X	X	X	X	X	X	X	X	X	X	X	X
***Record AE/SAE***	X	X		X^a^	X	X	X	X	X	X	X	X	X	X	X	X	X

## Sample size {14}

### Aims 1 and 2

#### Assumptions

Based on prospective data on HPV epidemiology in Africa, we anticipate 70% of young, sexually active women in Kenya will be HPV 16/18/31/33/45/52/58 naïve (as documented by baseline serology and HPV DNA) and eligible to contribute to an incident persistent HPV 16/18 and HPV 16/18/31/33/45/52/58 outcome. We assume the incidence of persistent HPV 16/18 to be 5 per 100 person-years and the incidence of persistent HPV 16/18/31/33/45/52/58 to be at least 5 per 100 person-years. We expect the efficacy of the bivalent and nonavalent vaccines to be 75% to prevent incident persistent HPV 16/18 infection due to lower geometric mean titers in observational data following single-dose versus three-dose vaccination [10]. To estimate the sample size, we assumed fixed follow-up time, i.e., all persons are followed for the same length of time, and loss-to-follow-up of 10%. The sample size estimate is based on a fixed number of events and the Hazard Ratio models (SeqDesign in R). For the null hypothesis, the HR = 1.0 and for the alternative hypothesis the HR = 0.25.

#### Total sample size

Accounting for women who are HPV 16/18 DNA-positive or seropositive at baseline, we estimate that 2250 young women age 15–20 years would need to be enrolled (750 participants per arm). We estimated, with a 3:1 screening to enrollment ratio, approximately 9000 young women will be screened to identify 2250 eligible participants.

#### Power

Accounting for 30% prevalent HPV infections at baseline and month 3, 10% loss-to-follow-up (LTFU), and 1-year fixed follow-up time after accrual, we have 90% power to see a 75% reduction in persistent infections between either of the two immediate HPV vaccine arms and the delayed HPV vaccination (Table 2, which shows the average number of events per arm). Assuming an additional 18% prevalent infections at month 3 for a total of 48% prevalent infections excluded at baseline and month 3, 10% LTFU, and 1-year fixed follow-up time after accrual, we have 80% power to see a 75% reduction in persistent incident infections between the immediate and the delayed vaccine arms.

**Table 2:** DSMB recommendations

Primary data analysis recommendation	Communication strategy probable components
1. That the study continues as planned.	Routine action indicated • Routine communication to PIs and critical collaborators • Distribution of primary data analysis minutes to relevant parties
2. That the study be modified because policy changes now recommend single-dose vaccination. 3. That the study be stopped due to concerns about participant safety, including social harms.	Urgent action indicated • Expedited communication to PIs, critical collaborators, regulators, and associates • Key messages and briefing materials • Protocol modifications • Participant information • IRB and regulatory approvals • Distribution materials to relevant organizations

#### Adjusting the timing of the primary analysis to ensure adequate power for the analysis

If the coronavirus disease (COVID-19) pandemic delays follow-up, the primary analysis could be delayed to allow sufficient endpoints and adequate power for the primary analysis.

## Recruitment {15}

### Strategies for recruitment

The study sites have experience recruiting young women for intervention studies including HPV vaccine studies, pre-exposure prophylaxis (PrEP) for HIV prevention studies, and sexually transmitted infection studies. We engage key stakeholders including cervical cancer prevention advocacy groups, non-governmental organizations, and local and national Ministry of Health staff. Study teams use informal baseline surveys at local training centers, clinics, non-governmental organizations, and colleges to identify where the study population will be recruited. Each site also identifies and analyzes the stakeholders in terms of their influence, level, and engagement mechanism. Using this information, the teams construct map recruitment areas. Recruitment approaches include the following strategies: engagement with identified health facilities or learning facilities attended by adolescents and young women, working with enrolled participants to identify and refer their peers who may be interested for further information (respondent driven sampling recruitment techniques), and conceptualize general messages into a drama for local radio stations to create awareness on HPV and the need for cervical cancer screening. Other strategies include the use of mobilizers, mission/community theatre recruitments, cervical cancer screening campaigns, and use of technology such as phones and tablets for information and education (edutainment) to maximize recruitment. The stakeholder information analysis is used to design community entry, mobilization, and engagement. All study related recruitment materials have regulatory approval.

## Assignment of interventions: allocation

### Sequence generation {16a}

#### Randomization procedures

The randomization allocation lists are generated using computer-generated random numbers to create blocked randomization, stratified by site. A 1:1:1 random allocation to the three arms of the trial was used. Randomization is blocked to ensure balance between the arms and stratified by site to account for potential different risks of incident persistent HPV infection. The randomization is designed by a study statistician or designee. A copy of the code used to generate the randomization, the block size used, and the randomization list itself are stored in a secure directory, to which only unblinded members of the KEN SHE statistical team have access.

## Concealment mechanism {16b}

Blinded study assignment is implemented via http://www.randomize.net, or when necessary due to lack of internet, using a back-up envelope system. The randomization arm is not determined until the participant has completed all screening procedures (above).

### Randomize.net

Unblinded members of the statistical team generated the allocation order for randomization and provided separate allocation lists to Randomize.net, as well valid participant IDs (PTIDs).

The randomization sequence is implemented in sequential order for each site. Unblinded site pharmacy staff enter the PTID on Randomize.net, the PTID is verified through Randomize.net, and the next sequential treatment arm is assigned. The date of randomization and the blinded “randomization ID” are witnessed on site and are recorded on the study source documentation and study case report form.

### Back-up randomization envelopes

A limited number of envelopes (*n* = 30 per site) are provided to sites for use in randomization allocation in the case of power or internet outage where the site is unable to access Randomize.net using a computer or a mobile phone. To create the back-up envelopes, additional blocks of randomization assignment were generated by the KEN SHE statistical team, and printouts that detail envelope number, randomized vaccine, and blinded randomization ID were inserted into each of 30 envelopes, with the sequence number on the printout matching that on the envelope. Envelopes are delivered to the sites at Site Activation Training.

### Vaccine masking

The study participants, investigators, site personnel, the laboratory staff conducting HPV typing of clinical samples by serology and PCR, and the laboratory staff who read and issue diagnoses of Pap tests are blinded to the vaccination group assignments of the subjects for the duration of the study. The site pharmacist is unblinded to the randomization arm and uses Randomize.net to obtain the study group, record the PID and randomization ID on a CRF, and draw up the vaccination for administration. The vaccine volumes are identical (0.5 ml), and the syringes appear identical. The syringe markings are concealed with opaque labels or tape and the syringe is labeled with the PID. The vaccine is administered after the PID is confirmed.

### Implementation {16c}

The enrolment visits is conducted by a blinded KEN SHE study team member.

### Randomization

Participants are randomly assigned 1:1:1 to immediate single-dose bivalent or nonavalent HPV vaccine (and delayed meningococcal vaccine) or delayed nonavalent HPV vaccine (and immediate meningococcal vaccine) by the unblinded study pharmacist, using the web-based randomization software.

### Vaccination

The vaccine information (lot number and expiry date) is recorded on pharmacy logs by the unblinded study pharmacist. The unblinded pharmacist accesses the web-based randomization or opens the randomization envelope in the numbered sequence and records the PID and blinded randomization ID on a CRF. The pharmacist is responsible for vaccine inventory and accounting, including vaccine that is not used, for example, if the dose is broken. Vaccines will be stored under appropriate controlled conditions with regulated access.

### End of the enrolment visit

At the end of the enrolment visit, an appointment is made at month 1 for substudy participants, and at month 3 for all participants. The first 900 participants were included in the immunobridging substudy. Staff provide information on adverse events associated with vaccination, ensured participants have contact information for the study team, and answer participant questions.

## Assignment of interventions: blinding

### Who will be blinded {17a}

The study participants, investigators, site personnel, the laboratory staff conducting HPV typing of clinical samples by serology and PCR, and the laboratory staff who read and issue diagnoses of Pap tests are blinded to the vaccination group assignments of the subjects for the duration of the study.

### Procedure for unblinding if needed {17b}

Participants may be unblinded in consultation with the Country Medical Director and Principal Investigators for clinical reasons. Participant may choose to be vaccinated outside the trial and continue follow-up in the study.

## Data collection and management

### Plans for assessment and collection of outcomes {18a}

#### Follow-up procedures

Participants complete a questionnaire on clinical history including adverse events, symptoms of sexually transmitted infections (STIs), exposure and sexual behavior, clinical health, updated contact information, and adherence to SRH services. A limited physical exam and pelvic examination for specimen collection is conducted every 6 months. Providers conduct visual inspection for genital warts and a speculum examination to collect a lateral vaginal swab and cervical swab for HPV DNA testing and specimens for STI testing. The procedures for the pelvic and speculum exams are explained to the participants, questions answered. If a participant declines pelvic examination, they are given the option to self-collect a vaginal swab.

Specimens for HPV DNA testing are collected by clinicians every 6 months. An additional swab is self-collected by the participant at month 3 for HPV DNA testing.

Serum is collected for HPV 6/11/16/18/31/33/45/52/58 serologic testing at enrollment, month 18 and month 36 for all participants and at month 1 and month 24 for the 900 immunobridging sub-study participants to test the immunologic response to the vaccines, as part of the immunobridging sub-study described below.

Sixty participants, consecutive participants at month 1, and 40 participants at months 36 and 37 will have ~ 20–40 mL of blood collected for B cell immunologic testing, to determine if the vaccine establishes long-term memory for neutralizing HPV infections. Once training for the B cell substudy is complete, enrollment of consecutive participants begins.

As part of comprehensive SRH services, contraception; STI, HIV, and pregnancy testing; and PrEP will be offered to participants every 3 months. Participants with new medical concerns will be referred for care as appropriate.

In response to the COVID-19 pandemic, telehealth visits were introduced for follow-up visits at months 9, 15, 21, and 27 to complete visit questionnaires. For visits with cervical swab collection, months 6, 12, 18, and 24, participants were also given the option to have the swab collected at a near-by facility or self-collect the swab for collection by courier. Contraception and PrEP delivery were also available by courier.

#### Laboratory assays

Specimens for HPV testing are transported overnight by courier at ambient temperature to the UW Mombasa Laboratory for HPV testing. If storage is necessary prior to courier pickup, the samples are maintained at 4°–25 °C for up to 4 weeks from collection date. To ensure temperature excursions do not occur, the specimen package is placed in a cooler box with ice packs to maintain appropriate temperature during transit. Specimens are not allowed to freeze. The temperature is confirmed by the receiving laboratory.

HPV DNA genotyping is conducted using the Anyplex II HPV28 assay (Seegene, Seoul, South Korea), a multiplexed real-time type-specific PCR assay that simultaneously detects, differentiates, and semi-quantifies 28 HPV genotypes (19 hrHPV types and 9 lrHPV types) [16, 17]. The Mombasa laboratory participates in proficiency studies for HPV DNA detection and typing.

Plasma or serum specimens are shipped to the University of Washington, Seattle, WA, USA, where they are scanned and stored for specialized antibody testing. At the time of testing, specimens are transported to the nearby (2 miles) Fred Hutchinson Cancer Research Center under controlled conditions. HPV antibodies are detected in serum or plasma using a Luminex assay for detecting HPV antibodies. For the subset of women in the B cell studies, peripheral blood mononuclear cells (PBMC) are prepared at the Kisumu Site Laboratory in Kenya and shipped on dry ice directly to the Galloway Lab at Fred Hutchinson Cancer Research Center.

#### Plans to promote participant retention and complete follow-up {18b}

Retention activities are proactive. Participants receive text messages or telephone calls reminding them of their upcoming study visits. At study follow-up visits, free comprehensive sexual and reproductive services are offered on site. Outreach is conducted by a study team member to participants who miss visits by text, phone, or in-person visits, with their permission.

The sites’ retention strategies focus on:

1. Personalized attention: counseling focused on individual adolescent girls and young women, listening, empathy, and proactive management such as linking to appropriate services,
2. High quality standard of clinical care through regular staff training,
3. Comprehensive SRH services,
4. Good customer care with a holistic approach including baby friendly clinics, and
5. Participant event days to celebrate girls and young women and provide learning opportunities.

## Data management {19}

### Data collection

Completed electronic cases report forms (CRFs) and laboratory forms are submitted to the data analyst at the University of Washington. Data are entered into a study-specific database by trained study staff using the electronic DFnet© software.

The primary mode of data collection for case report forms is electronic, with paper forms as backup in case of internet outages. Data collected on paper CRFs are entered electronically once connectivity is reestablished. The computers used by clinicians and data personnel are password-protected. Source documents such as some laboratory results are in paper format.

### Data quality checks

Data quality control is done on-site. Additional data checks and data cleaning are done by trained data managers at DFnet and UW under the supervision of a senior data manager. Study progress is monitored weekly with either fortnightly, or, at a minimum, monthly quality control measures conducted by the study analyst, based at the University of Washington, with oversight from the senior data manager and study investigators.

### Data management plan

When reviewing, exporting, or managing data, all communications between browser and server are encrypted. Servers are secured by firewalls to prevent unauthorized access. Data is protected from virus threats using anti-virus technology. The study database is backed-up regularly, both on-site and off-site. At the conclusion of the study, the database will be archived in accordance with study site procedures. Data captured on paper forms are stored securely in locked file cabinets.

Site investigators maintain, and store in a secure manner, all study records throughout the study implementation and post implementation. All study clinics have an archival store with limited access and fire safety measures in place. All study records will be retained according to local regulatory guidelines.

## Confidentiality {27}

Site staff conduct all study procedures in private and protect participant privacy and confidentiality to the extent possible. Study-related information is stored securely at the sites. Sites maintain any records that contain names or other personal identifiers, such as locator forms and informed consent forms, separately and securely with limited access. Sites also secure forms, lists, logbooks, appointment books, and any other listings that link participant numbers to identifying information in a separate, locked file area. Laboratory specimens, data collection, and administrative forms are identified only by coded number and are also kept secure, with access limited to study staff. Sites will protect any on-site databases with password access systems.

Participants’ study information will not be released without their written permission, except as necessary for review, monitoring, and/or auditing by the following:

- KEMRI Scientific Ethics Research Unit (SERU)
- Kenya Pharmacy and Poison Board
- Representatives of the US Federal Government, including the US OHRP, representatives of the US and host government and other local and US regulatory authorities
- Study management staff
- Site staff
- Site and central IRBs/ECs
- Sponsors
- Contractors working on behalf of the trial management

## Plans for collection, laboratory evaluation, and storage of biological specimens for genetic or molecular analysis in this trial/future use {17}

Specimens collected in this trial are stored for future use. Blood and genitourinary specimens are stored securely at the Thika, Nairobi, and Kisumu sites and at the University of Washington and the Fred Hutchinson Cancer Research Center for future research. These samples may be used for research related to HPV vaccines, HPV, BV, HIV, HIV-related diseases, and STIs. Samples are stored using barcodes that are not personal identifiers. The Scientific and Ethics Review Unit (SERU) in KEMRI, Kenya, has regulatory oversight over the safety and rights of research participants and must approve any future research studies using study data and samples. Participants can decline storage of samples when completing their informed consent or withdraw consent for sample storage up to 5 years after the study conclusion.

## Statistical methods

### Statistical methods for primary and secondary outcomes {20a}

#### General statistical considerations

Descriptive analyses summarizing baseline and follow-up data will be stratified by site and/or country and treatment arm separately. Line, scatter, and box plots will be used, as appropriate, for longitudinal data representations. If loss to follow-up is greater than 10%, baseline characteristics will be described by loss to follow-up status. Missing data will be imputed.

## Cohorts and datasets for primary and supporting analyses

Analyses will be performed on the following populations.

### Intent to treat cohort

The intent to treat (ITT) population includes all enrolled participants

### Modified intent to treat cohort

The modified intent to treat (mITT) cohort is defined as participants who are HPV antibody negative at the enrolment visit and HPV DNA negative from the enrolment visit to (and including) the month 3 visit. Participants who are HPV DNA positive at month 3 are excluded to ensure exclusion of all participants who had incubating infection and were not yet PCR positive.

### Modified intent to treat sensitivity cohort

The mITT Sensitivity cohort differs from the mITT cohort in that participants are only excluded based on the HPV DNA test and not HPV antibody tests. Therefore, the modified intent to treat (mITT) sensitivity cohort is defined as participants who are HPV DNA negative from the enrolment visit to (and including) the month 3 visit. Participants who are HPV DNA positive at month 3 are excluded to ensure exclusion of all participants who were infected at enrolment.

### Extended sensitivity cohort

The extended sensitivity cohort is defined as the analysis cohort for participants who are HPV DNA negative from enrolment to (and including) the month 6 visit. Participants who are HPV DNA positive at month 6 are excluded to ensure exclusion of all participants who were infected at enrolment and for consistency with previous studies [18].

## Primary efficacy analysis

### Objectives

1. To separately test the efficacy of immediate single-dose bivalent and nonavalent HPV vaccination to prevent incident persistent HPV 16/18 infection compared to delayed nonavalent HPV vaccination for young women age 15–20 years.
2. To test the efficacy of immediate single-dose nonavalent HPV vaccination to prevent incident persistent HPV 16/18/31/33/45/52/58 infection compared to delayed nonavalent HPV vaccination for young women age 15–20 years.

### Outcome

Outcome is as follows: Persistent HPV, defined as vaccine type specific HPV detected at two consecutive time points no less than 4 months apart after month 3 and up to and including 18 months apart (same HPV type at each time point) in the first 18 months post-randomization.

### Cohorts

Cohorts are as follows: mITT cohort, mITT sensitivity cohort, and extended sensitivity cohort.

### Definition of endpoints

The persistent HPV endpoints (16/18 and 16/18/31/33/45/52/58) are defined in participants who experience the outcome defined above as the time from the month 3 visit to first detection of vaccine type specific HPV.

### Analysis approach

The primary analysis will be performed on the mITT Cohort. If the participant does not reach the outcome of persistent oncogenic HPV infection, as defined above, her infection time will be censored at the last negative test date at or before the month 18 visit (time from month 3 visit to last negative test). A Cox proportional hazards (PH) model stratified by site will be used to assess the efficacy of each vaccine arm compared to control. Vaccine efficacy will be expressed as a percent reduction in the incidence rate (i.e., 100 × [1 − (vaccine infection rate/corresponding control infection rate)]). The log rank test stratified by site will be used to calculate the *p* value. Further details for the analysis are available in the statistical analysis plan.

## Secondary efficacy analyses

1. Immunobridging sub-study:

Sample size calculation: Using the same assumptions as the main study, we anticipate 70% of young, sexually active women in Kenya will be HPV 6/11/16/18/31/33/45/52/58 naïve (as documented by baseline serology and HPV DNA).

Total sample size: Using the same assumptions as the main study, we estimate that 300 women from each arm (300 × 3 = 900 total) would need to be enrolled in the sub-study.

Power: When the sample sizes in the groups are 150 and 300, a two group 0.025 one-sided *t* test will have 91% power to reject the null hypothesis that the test is inferior to the standard in favor of the alternative hypothesis that the treatment is non-inferior, assuming that the expected difference in log means is 0 (i.e., the ratio is 1.0), a non-inferiority margin of log (0.67) and the common standard deviation is 1.2.

Immunobridging analysis:

Bivalent vaccine: The cohort for primary analysis of non-inferiority includes 15–20-year-old women from this trial and 9–14-year-old girls from the Tanzania trial who received single-dose HPV 16/18 vaccination and who were HPV 16/18 DNA negative and HPV 16/18 seronegative at baseline [19].

Nonavalent vaccine: The cohort for primary analysis of non-inferiority includes 15–20-year-old women from this trial and 9–14-year-old girls from the Tanzania trial who received single-dose HPV 6/11/16/18/31/33/45/52/58 vaccination and who were HPV 6/11/16/18/31/33/45/52/58 DNA negative and HPV 6/11/16/18/31/33/45/52/58 seronegative at baseline [19].

For each vaccine group (bivalent and nonavalent), noninferiority of antibody GMTs at 1 month and 24 months after single-dose vaccination will be tested using a 2-sided 95% CI for the ratio of antibody GMTs in 9–14-year-old girls relative to antibody GMTs in 15–20-year-old young women. Noninferiority will be tested for each vaccine type. Noninferiority will be declared if the lower limit of the ratio is above 0.67. The statistical criterion for non-inferiority requires that the lower bound of two-sided 95% confidence interval of GMT ratio (girls vs. young women) be greater than 0.67 for each HPV type. To facilitate robust analysis of immune-bridging and account for the durability of vaccine efficacy, the 24-month analysis will be the primary analysis with the month 1 analysis providing additional information.

## Analysis plan for B cell studies

For the bivalent and nonavalent vaccine vs. the meningococcal vaccine and for the bivalent vs. nonavalent vaccines we will compare (1) the percent of plasmablasts from PBMCs, (2) the percent of plasmablasts from purified B cells, (3) the percent of HPV 16 specific B memory cells, and (4) number of HPV 16 B memory cells per 10^6 live cells. We will use the ANOVA test for pairwise comparison and the ANOVA for more than two groups. Differences with a *p* value of < 0.05 will be considered significant.

## Cost-effectiveness analysis

Cost effectiveness analysis: We will assess cost, cost-effectiveness, and budget impact of single-dose HPV vaccination to support implementation strategies for single-dose HPV vaccination following WHO recommendation in high cervical cancer burden settings.

Overall approach: Using activity-based micro-costing data and outcome data from aim 1, we will define the costs and model the cost-effectiveness of single-dose HPV vaccination for optimized cervical cancer prevention. We will work closely with the Kenyan Ministry of Health to determine the package of evidence necessary for implementation of single-dose HPV vaccination including budget impact and strategies for mass single-dose vaccination and incorporation of HPV vaccination into health services for young women.

Health economic modeling: We will adapt our existing South African HPV transmission model to explicitly include single-dose bivalent and nonavalent HPV vaccination and parameterize a transmission model for the Kenyan HPV transmission and progression to cervical cancer [20].

*Model outcomes*: We will estimate the impact of single-dose bivalent and nonavalent HPV vaccination on the change in (1) cervical cancer incidence, (2) cervical cancer related deaths, and (3) disability-adjusted life years (DALYs). The model simulates the intervention impact and projects the effect on health outcomes over 10 years. For all key inputs and outputs, we will follow standard practices [21], including the guidelines by the Panel of Cost-Effectiveness in Health and Medicine [22]. We will report on all costs using a recommended discount rate of 3% per year, as well as an alternative 5% discount rate and undiscounted inputs.

## Sensitivity analyses

The analyses will be repeated on the mITT sensitivity cohort and the extended sensitivity cohort. The analyses will be repeated on the ITT cohort as an exploratory analysis.

### Durability analysis

These final analyses will be the same as the primary analysis (including sensitivity analyses) with the outcome defined until 36 months instead of 18 months.

### Interim analyses {21b}

Interim analyses are not planned for the study.

## Methods for additional analyses (e.g., subgroup analyses) {20b}

### Subgroup analyses

Analyses will be conducted for the following prespecified subgroups based on baseline covariates: presence of co-infections (chlamydia, gonorrhea, herpes simplex, trichomoniasis, syphilis, bacterial vaginosis), self-reported condom use, number of self-reported sex partners in the last 3 months (0–1 vs. 2+), contraception method, and PrEP use.

## Methods in analysis to handle protocol non-adherence and any statistical methods to handle missing data {20c}

### Missing data

Some participants may have a single positive HPV DNA test without another test in the required timeframe to determine persistent HPV. The primary efficacy analysis above will be repeated twice: once assuming all missing tests would have been positive and a second assuming all missing tests would be negative.

## Plans to give access to the full protocol, participant-level data, and statistical code {31c}

The full protocol will be made available. Data cannot be shared publicly because this study was conducted with approval from the Kenya Medical Research Institute (KEMRI) Scientific and Ethics Review Unit (SERU), which requires that data from studies (including de-identified data) are released only after SERU have provided written approval for additional analyses. A complete de-identified dataset sufficient to reproduce the study findings will be made available upon written request after approval from SERU. To request these data, please contact the KEN SHE Scientific Committee at icrc@uw.edu.

## Oversight and monitoring

### Composition of the coordinating center and trial steering committee {5d}

Our multi-disciplinary University of Washington (UW)-Kenya Medical Research Institute (KEMRI) Coordinating Center team consists of clinical trialists (Drs. Barnabas, Mugo, Winer, Bukusi, Baeten, and Celum), HPV experts (Drs. Winer, Galloway, Mugo, Barnabas), biostatisticians (Drs. Brown, Donnell), an immunologist (Dr. Galloway), obstetrician gynecologists (Drs. Mugo and Bukusi), and an economic modeler (Dr. Barnabas) who have experience with translational science and implementation. The Coordinating Center capacity is strengthened by collaborative work between scientific operations and implementing partners. The co-PIs and investigators have weekly calls to track the study progress and ensure fidelity to the study design, timeline, and budget. An endpoint adjudication committee (Drs. Barnabas and Winer) will meet to review study endpoints.

### Composition of the data monitoring committee, its role and reporting structure {21a}

#### Data Safety and Monitoring Board review for safety and study execution

The Data Safety and Monitoring Board (DSMB) is independent and consists of six members: global expert clinical trial statistician, clinical trialists, experts in adolescent health including HPV infection, experts in cervical cancer treatment and prevention, and policy experts. The DSMB meets every 6 months to review the available study data. The DSMB evaluates participant safety (SAEs and social harms), available endpoint data, and reviews the operational factors, specifically participant enrollment and follow-up, to assess safety, study execution, and provide feedback for investigators on areas for attention.

#### DSMB stopping rules

We will plan a formal primary analysis to evaluate study outcomes after 18 months of follow-up. We will follow the policy recommendations for single-dose vaccination, given the expected emerging evidence from this and other clinical trials. Both the magnitude and durability of the effect through 36 months of the single-dose HPV vaccine are critical outcomes of this study.

## Adverse event reporting and harms {22}

The three sites monitor adverse events (AEs) according to the local guidelines of the KEMRI’s Scientific and Ethics Regulatory Unit (SERU), which is the site local regulatory authority. Adverse events are reported in the study data on eCRFs. Additional information about these AEs, including copies of outpatient records, hospitalization summaries, pathology reports, operative reports, and laboratory reports, are included in the reports as applicable. The SERU reporting process includes reporting all study-defined SAEs to SERU via email (seru@kemri.org) within 48 h after the PI (or official designee) becomes aware of the event. The hard copies of the report are forwarded to the SERU Secretariat within three working days of the initial notification. Follow-up reports are submitted as soon as more information becomes available. The written safety report is addressed to the SERU Chairperson and submitted to the SERU Secretariat. The reporting includes the PI’s (or official designee’s) opinion on the relationship of the adverse event with participation in the study.

## Social harm reporting

Although study sites make every effort to protect participant privacy and confidentiality, it is possible that participants’ involvement in the study could become known to others and that social harms may result. The three sites assess for study-related social harm at each scheduled follow-up visit; reports can also be filed at interim visits if study-related social harms are discovered between visits. Information regarding social harms related to study participation is recorded into the study database. Participants who report social harms are referred to speak with a study counselor and, if appropriate, a study clinician and the site investigator/designee. The three sites refer participants to appropriate additional resources for safety as needed. If the site investigator/designee judges a social harm related to study participation to be serious and unexpected, the study safety monitor and SERU is notified within 10 days of site awareness.

## Frequency and plans for auditing trial conduct {23}

The trial is being monitored by an independent study monitor every six months, which includes 100% quality checks of the vaccine randomization.

## Plans for communicating important protocol amendments to relevant parties (e.g., trial participants, ethical committees) {25}

Prior to implementation of this protocol, and any subsequent full version amendments, the three sites had the protocol and the protocol consent forms approved by SERU and Poisons and Pharmacy Board (PPB). Prior notice was given and written consent was sought and obtained from all study participants. All study participation is strictly voluntary, and participants can refuse specific procedures, or further study participation at any time.

## Protocol amendments

If changes are needed to the protocol, the protocol implementation team (in accordance with the protocol design team) handles all changes centrally. Once the changes are finalized, the protocol is submitted and approved by the appropriate IRBs and regulatory agencies prior to implementation. Version control is maintained centrally and sites will be trained on any changes.

## Dissemination plans {31a}

Our team of investigators is fully committed to rapid and multi-pronged dissemination of study results, regardless of the results. Through our previous HIV prevention trials (Partners in Prevention HSV/HIV Transmission Study, Partners PrEP Study, ASPIRE Study [23–25]), we have found that community and stakeholder consultations are important at every stage of the research, spanning study concept to results, in order to build and sustain trust in research and research partnerships. Once results are available, we will provide timely results to the Kenyan Ministry of Health, community stakeholders, and provide data for the SAGE HPV vaccine meeting. Once study analysis is complete, data will be presented at local and international conferences and will be submitted for publication.

The three sites will carry out dissemination meetings in Kenya bringing together international, regional, and local stakeholders to discuss findings and their implications for national policies. Since the results may directly influence national policies and recommendations, results will also be shared and discussed with the National Kenya Division of Family Health, Vaccine Program, and stakeholders’ forum and the Adolescent Sexual Reproductive Health Technical Working Group of which the site PI (NM) is a member. At the local level, the results will be shared and discussed with the County specific Kenya Division of Family Health, Vaccine Program, and Adolescent Working Groups of which KEMRI is represented.

## Discussion

This randomized, double-blind, controlled trial will provide data on single-dose HPV vaccine efficacy among adolescent girls and young women age 15–20 years. While observational evidence supports the efficacy of single-dose HPV vaccination [8, 10, 12], this study aims to provide estimates of efficacy to guide public health policy. Further, observational evidence supports multi-age cohort vaccination, up to age 26 years in high-income countries, as a key driver to prevent moderate and severe precancerous lesions over the shortest time period [6]. This strategy of multi-age cohort vaccination has been limited in low-and-middle-income countries by the high cost of the HPV vaccines, the limited supply, and the recommendation for three doses among persons older than 15 years. The KEN SHE Study aims to close that gap in evidence.

The final results of the KEN SHE study are expected in early 2023 and the primary results in Q1 2022. Other trials evaluating single-dose protection are ongoing in Costa Rica (the ESCUDDO trial; NCT03180034), the Gambia (the HANDS trial; NCT03832049), and Tanzania (the DoRIS trial; NCT02834637), with results available at the end of 2021 or later [9, 19]. These trials are all complementary, examining single-dose HPV vaccination for girls, adolescents, and young women age 4–20 years and address different scientific and programmatic questions. In addition to comparing efficacy between study groups, antibody results from A Dose Reduction Immunobridging and Safety Study of Two HPV Vaccines in Tanzanian Girls (DoRIS) Study will be used to immunobridge to the antibody results to the KEN SHE Study (by demonstrating non-inferior antibody levels) enabling extension of the single-dose approach down to girls age 9–14 years [19].

The study conduct has encountered and addressed challenges raised by the COVID-19 pandemic. During the global coronavirus (COVID-19) pandemic, as of May 17, 2021, Kenya has reported 165,465 cases, 3003 COVID-19 deaths, and a current case fatality rate of 1.8% [26]. Approximately 7000 SARS-CoV-2 tests were conducted daily in February 2021. Participant research visit were restricted in March and April 2020, but reopened in May 2020. Additional restrictions were in place in April 2021 with a third COVID-19 wave but these were lifted in May 2021 with a decrease in cases. Emergency services were available to participants throughout. WhatsApp discussion groups were started to facilitate participant retention, and visit questionnaires were completed by phone, with specimen collection resuming once SERU guidelines for in-person visits were in place. Clinical trial sites instituted COVID-19 safety precautions, following SERU guidelines, including symptom screening for participants and staff by phone prior to arrival at the clinic, temperature checks at the clinic, use of personal protective equipment, and social distancing where possible. Travel and in-person learning have re-opened with safety precautions to prevent SARS-CoV-2 transmission.

The KEN SHE Study data will contribute to the Global Strategy towards Eliminating Cervical Cancer as a public health problem led by the World Health Organization (WHO) [27]. Cervical cancer is the 4th most common cancer among women globally, and, the 2nd most frequent in sub-Saharan Africa (SSA), mostly affecting women between ages 30–49 years, and the leading cause of new cancer deaths in SSA [28, 29]. Cervical cancer is an almost entirely preventable cancer with HPV vaccine and the most curable form of human cancer with early detection and treatment. In August 2020, the WHO adopted the Global Strategy to eliminate cervical cancer by scaling up prevention through HPV vaccination—with the global target to reach 90% coverage of HPV vaccination by 2030, 70% coverage of screening and treatment of pre-cancerous lesions, and 90% coverage of management of invasive cervical cancer [27]. However, the estimated global coverage among 9–14-year-old girls for HPV vaccination is 40% with only 8–9% of 10–20 year olds vaccinated [30]. Further, HPV vaccine supply is constrained [7] and SAGE has called for options to optimally allocate existing HPV vaccine supply. Effective single-dose HPV vaccination would facilitate rapid scale up of vaccination worldwide or, in the absence of public health benefit, redouble efforts to ensure adequate supply and implementation of a two- or three-dose vaccination strategy to meet the goal of cervical cancer elimination.

The trial has several strengths including the randomized controlled blinded design, testing two vaccine types, and testing vaccine efficacy over a short time frame to support policy decisions. Further, the secondary objectives will evaluate immunobridging to younger trial participants, assess central memory for vaccine durability, and estimate the population-level impact on cervical cancer incidence and cost-effectiveness, building on the primary study results. A potential limitation of the study is not recruiting participants prior to sexual debut which may limit the number of HPV naïve participants eligible for assessment of vaccine efficacy.

In summary, the KEN SHE Study will evaluate single-dose HPV vaccination and provide robust estimates of vaccine efficacy against persistent HPV infection among adolescent and young women in sub-Saharan Africa. These data combined with other ongoing trials will provide evidence for policy makers on strategies to increase HPV vaccine coverage as a key component of cervical cancer elimination interventions.

## Trial status

Protocol version 1.0, November 15, 2018

Protocol version 2.0 includes the provision to delay analysis with COVID-19 delays in study procedures and clarifies the primary and secondary objectives.

Protocol version 2.0, January 7, 2021.

Participants were recruited between December 20, 2018, and November 15, 2019. Participants will continue follow-up in the study until December 2022, with analysis and dissemination of results in Q1-2 in 2023. Protocol submission was delayed due to the COVID-19 pandemic.

## Supplementary Information

**Additional file 1.** SPIRIT 2013 Checklist: Recommended items to address in a clinical trial protocol and related documents.

## Data Availability

Data cannot be shared publicly because this study was conducted with approval from the Kenya Medical Research Institute (KEMRI) Scientific and Ethics Review Unit (SERU), which requires that data from studies (including de-identified data) are released only after SERU have provided written approval for additional analyses. A complete de-identified dataset sufficient to reproduce the study findings will be made available upon written request after approval from SERU. To request these data, please contact the KEN SHE Scientific Committee at icrc@uw.edu.

## Abbreviations

AE: Adverse event
BV: Bacterial vaginosis
CAB: Community advisory board
CI: Confidence interval
CRF: Case report form
DALY: Disability-adjusted life year
DoRIS: A Dose Reduction Immunobridging and Safety Study of Two HPV Vaccines in Tanzanian Girls (DoRIS)
DNA: Deoxyribonucleic acid
DSMB: Data Safety and Monitoring Board
EPI: WHO Expanded Program on Immunization
FDA: US Food and Drug Administration
GAVI: Global Alliance for Vaccines and Immunization
GCP: Good Clinical Practice
GCLP: Good Clinical Laboratory Practice
GMT: Geometric mean titer
HIV: Human immunodeficiency virus
HSV-2: Herpes simplex virus type 2
HPV: Human papillomavirus
hrHPV: High-risk human papillomavirus
IARC: WHO International Agency for Research on Cancer
ITT: Intention-to-treat
KEMRI: Kenya Medical Research Institute
KEN SHE: KENya Single-dose HPV-vaccine Efficacy (KEN SHE) Study
LMP: Last menstrual period
lrHPV: Low risk human papillomavirus
LEEP: Loop electrosurgical excision procedure
LTFU: Loss to follow-up
NCI: US National Cancer Institute
NIH: US National Institutes of Health
PBMC: Peripheral blood mononuclear cells
PCR: Polymerase chain reaction
PrEP: Pre-exposure prophylaxis
RTI: Reproductive tract infection
SAE: Serious adverse event
SAGE: Strategic Advisory Group of Experts on Immunization
SOP: Standard operating procedure
SRH: Sexual and reproductive health services
SSA: Sub-Saharan Africa
STI: Sexually transmitted infection
UW: University of Washington
VLP: Virus-like particle
WHO: World Health Organization
